# The effects of saline water consumption on sperm parameters, testicular histopathology, hormonal and antioxidants concentrations in Barki Rams

**DOI:** 10.1186/s12917-024-04047-2

**Published:** 2024-05-23

**Authors:** Rasha S. Mohamed, Ragab H Mohamed, Axel Wehrend, Enas A. Tahoun, Hassan A. Hussein

**Affiliations:** 1 Department of Animal Health, Animal and Poultry Production Division, Desert Research Center, Cairo, Egypt; 2 Department of Theriogenology, Faculty of Veterinary Medicine, Aswan University, Aswan, 81528 Egypt; 3 Clinic for Obstetrics, Gynecology and Andrology of Large and Small Animals with Veterinary Ambulance, Justus-Liebig University, Giessen, Germany; 4 Department of Pathology, Faculty of Veterinary Medicine, University of Sadat City, Menofia, 32897 Egypt; 5 Department of Theriogenology, Faculty of Veterinary Medicine, Assiut University, Assiut, 71526 Egypt; 6 Faculty of Veterinary Medicine, Sphinx University, New Assiut, Egypt

**Keywords:** Ram, Saline water, Reproduction, Antioxidants, Climatic change, Dryness

## Abstract

The study aimed to assess the effects of water salinity on the sperm parameters, levels of cortisol, LH, FSH, testosterone and antioxidants as well as the testes’ histopathology in Barki rams. Fifteen healthy Barki rams (1–1.5 years) were divided into three equal depending on the type of drinking water for nine months. The rams in the tap water group (TW, water that contained 350 ppm of total dissolved salts (TDS). Males in the high saline water group (HSW) were permitted to consume high saline water with 8,934 ppm TDS, whereas those in the second group were permitted to have moderately saline water (MSW, 4,557 ppm TDS). High salt concentration in drinking water had adverse effect on sperm viability, morphology and sperm cell concertation. Nitric oxide and malondialdehyde concentrations in blood were significantly higher in the MSW and HSW groups than in TW. There was a significant decrease in glutathione concentration as well as superoxide dismutase activity in TDS and HSW. Cortisol was most highly concentrated in the HSW, next in the MSW, and least in TW. The testosterone, LH, and FSH concentrations in the HSW and MSW groups were significantly lower than in TW. As the salt concentration in drinking water increases, damage to testicular tissue. The MSW group demonstrating vacuolation of lining epithelial cells with pyknotic nuclei in the epididymis and necrosis and desquamation of spermatogenic cells in seminiferous tubules while HSW group displaying desquamated necrotic cells and giant cell formation in the epididymis, as well as damage to some of the seminiferous tubules and showed congestion, vacuolation of spermatogenic epithelium of seminiferous tubules, and desquamated necrotic spermatogenic epithelium. In conclusion, the salinity of the water has detrimental impacts on the sperm morphology, viability and concentration, hormones and antioxidant levels in Barki rams.

## Introduction

Egypt’s agricultural land is only found in the Nile Valley and Delta, with few oases and a small amount of arable land in Sinai. Only 3% of Egypt’s land area, or 7.2 million Feddans, is under cultivation. One Feddan is equal to 4200 Km2 [1]. Small ruminants are more important than large ruminants for arable land to combat population exodus [2]. The contribution of livestock farming to rural families’ social and economic development is crucial [3]. Additionally, Small ruminants are the most commonly reared farm animals, and their production is significant for guaranteeing food security [4]. Breeding small ruminants provides a source of income and employment for rural residents, so it assists in reducing poverty and improving overall household well-being [5, 6]. Furthermore, to improve livestock production, it is critical to understand the various factors that influence stockbreeding [7]. Sheep and goats are vital sources of sustenance for villagers and are crucial in developing environmentally sound production systems [8]. The reason for the emphasis is that small ruminants hold a unique position in smallholder agriculture due to their shorter production cycles, faster growth rates, ease of management, low investment capital, low risk of loss, low feed requirements, and greater adaptability to harsh environmental climates than large ruminants [9, 10]. Unfortunately, little is known about small ruminant livestock production in Egypt’s rural areas [11]. A scarcity of food and water distinguishes deserts. This is complicated further during the long dry season by the frequently high salinity of drinking water obtained from wells in the North-Western Egyptian coastal desert, which can reach 10,797 ppm TDS [12]. According to Ray [13], water salinity is an essential factor in determining the suitability of a particular source for livestock. Animals living in these areas are also subjected to additional stressors, such as summer heat and cold during winter nights.

In humans, it was reported that producing reactive oxygen species (ROS) is one of the hypothesized processes by which a diet heavy in salt causes hypertension [14]. Oxidative stress, caused by ROS produced beyond the capacity of exogenous or cellular antioxidants, has been shown to impact the reproduction process [15, 16]. It has been observed that 25–40% of infertile men’s semen has elevated ROS levels [17] and that their semen has decreased antioxidant levels [15, 18, 19].

Domestic drinking water should have a TDS concentration of 400 ppm, and anything higher than that is deemed saline, according to recommendations from the public health service [20]. Freshwater scarcity is the principal barrier to adequate sheep production in Egypt’s arid South Sinai region [21]. Previous local investigations looked at how desert animals’ performance and physiological reactions were affected by water salinity [21].

The influence of water salinity on the testes’ histopathological appearance and the concentration of sexual hormones and oxidative stress status in rams is a subject of limited and contentious research. So, the current study aimed to ascertain how the saline water affected the amounts of LH, FSH, and antioxidant concentration in Barki Rams and the testes’ histological appearance.

## Materials and methods

### Animals and experimental design

All animal handling procedures, as well as sample collection and disposal, were performed in accordance in line with the regulations of the Institutional Animal Care and Use Committee (IACUC), Faculty of Veterinary Medicine, University of Sadat City, Egypt (Approval No. VUSC-03-7-22).

This study employed fifteen Barki Rams from the Animal Health Desert Research Center in South Sinai Peninsula, Egypt, with initial body weights of 32.30 ± 0.41 kg and ages of 1-1.5 years old. The experiment was carried out over nine months, and the ambient temperature, hum-index and relative humidity during the experiment were presented in Table (1) as the data obtained from https://www.worlddata.info/africa/egypt/climate-sinai-peninsula.php. The rams were divided into three equal subgroups (*n* = 5), each balanced for age and live body weight. The control group, designated as the tap water (TW) group, was permitted to consume tap water with 350 ppm TDS. The high saline water (HSW) group was allowed to drink high saline water with 8,934 ppm TDS (Table 2).

**Table 1:** The ambient temperature, hum-index and relative humidity during the experiment. (Source: https://www.worlddata.info/africa/egypt/climate-sinai-peninsula.php)

Parameter	Sep.	Oct.	Nov.	Dec.	Jan.	Feb.	Mar.	Apr.	May
Temperature (°C)	31.7	28.1	23.2	18.4	16.6	18.6	21.6	25.8	29.7
Relative Humidity (%)	36	40	46	52	51	45	38	32	29
Hum-index (°C)	39	35	29	24	21	23	26	31	35

**Table 2:** Water source in south Sinai research station and water analysis in the HSW (high saline water; 8,934 ppm of total dissolved salts), MSW (moderate saline water; 4,557 ppm of total dissolved salts) and TW (control/tap water; 350 ppm of total dissolved salts) groups

Salinity degree	Water source	TDS (mg/l)	Cations (mEg/l)				Anions (mEq/l)		
			Ca^++^	Mg^++^	Na^+^	K^+^	HCO3^−^	Cl^−^	SO4^−^
TW	Tap water	350	37.1	15.0	28.2	7.9	170.0	16.6	55.2
MSW	Moderate saline ground water well	4.557	216.4	86.9	1.232	15.6	323.4	1.386	1.287.2
HSW	High saline ground water well	8.934	386.7	167.8	2.416	35.0	457.6	3.301	1.858.8

TDS: Total dissolved solid; TW (Tap water); MSW (Moderate saline water); HSW (High saline water)

In comparison, the moderately salty water (MSW) group was permitted to consume rather salty water with 4,557 ppm TDS. The water offered to the rams was taken from tap water and underground saline, and it was categorized by the salinity level as shown in Table (1), and the access to water was *ad-lib*. Groups were kept apart in pens that provided shade. Before enrolling in the study, the males underwent a clinical examination to ensure their health. The SC anthelmintic injection [Ivermectin + Clorsulon 0.2 mg/kg Bwt (Ivomec® super in a dose of 1 ml/50 kg SC)] was used to deworm the rams twice, two weeks apart. The trial started after two weeks of acclimatization for the males. The Agriculture Research Council grant was used to determine the maintenance ration given to the test animals. Berseem hay (*Trifolium alexandrinum*) and concentrated mixture cubes formed of unadorned cotton seed (50%), rice polish (11%), wheat bran (18%), yellow maize (15%), molasses (3%), limestone (2%) and salt (1%), made up the ration. Food was provided every day at 8:00 a.m., and water was freely available for one hour while food was being consumed. The amount of feed given was decided based on maintenance requirements estimated locally. Throughout the trial, the rams were kept in a clean, well-ventilated stable under the same climatic, dietary, and hygienic circumstances. At the start of the trial and nine months following the saline water treatment, live body weight was measured. Table (2) shows the analysis and the water sources used in the experiment.

### Clinical examination

The sheep’s health was monitored at the beginning of the experiment by measuring body temperature, respiratory and pulse rates, ruminal movement, and mucous membrane condition to confirm the clinical soundness of the animals.

### Semen collection and evaluation

Using a clean and sterile artificial vagina, one semen ejaculate was collected per week from each ram. The rams were allowed to one false mount before semen collection, and then the ejaculate was collected during the subsequent mounting. Semen was kept at 37 °C and evaluated immediately upon collection for some parameters, including motility, viability and morphology of the spermatozoa. The mass motility of the spermatozoa was assessed at a low magnification (10×) and scored on a scale from 0 (no motility) to 5 (excellent motility). The percentages of live and dead sperms were determined from fixed-smear stained with eosin. Two hundred sperms were calculated from different fields in the stained smear; the colored head sperm was calculated as a dead sperm, while a colorless sperm was considered as a live sperm. The total sperm abnormalities were determined [22]. Sperm concentration was determined using the Hemocytometer with a triple line. Semen samples were fixed in formalin diluent [50 g sodium bicarbonate, 10 mL 35% (v/v) formalin in 1 L water] using the following dilutions: 1 semen + 1 formalized water [23]. A drop of semen suspension diluted with formalized water was placed under the hemocytometer cover slide and left for settling. Spermatozoa were counted in five large squares (one in each corner and one in the center) and then multiplied in dilution rate and number & volume of squares to obtain the sperm concentration per ml.

### Blood sampling and serum biochemical and hormonal analysis

Rams were given saline water daily for nine months. All sheep had their blood extracted through the jugular vein, and 5 ml of the blood was collected in a dry, clean vacuum tube (Biomedica Alex.Co., Alexandria, Egypt). Clear, non-hemolyzed serum samples were held at -20 °C until further examination after blood was clotted at room temperature for 20 min and centrifuged at 1008 g for 10 min. The serum samples were used to measure nitric oxide (NO), malondialdehyde (MDA), glutathione (GSH), and superoxide dismutase (SOD) using commercial kits provided by Spectrum Diagnostics (Obour City, Cairo, Egypt). The Clinical Chemistry Analyzer ERBA CHEM 7, ERBA (Mannheim, Germany) was used. The FSH levels were measured using an enzyme-linked immunosorbent ELISA kit (MyBioSource, Southern California, San Diego, USA) [24]. LH concentration was assessed using an ELISA kit (MyBiosourse, Southern California, San Diego, USA), while testosterone concentration was evaluated using an ELISA kit (MyBiosourse, Southern California, San Diego, USA) [25]. The cortisol concentration was assessed using the RIA method [26].

### Collection of tissue

At the end of the experiment, animals were slaughtered. Animals were fasted for 12 h with ad libitum access to water and transported to the slaughterhouse. An experienced technician conducted the slaughtering process by severing the jugular vein with a sharp knife without electrical stimulation or an aesthesia (By Islamic law, for further consumption of the carcass meat). The death of the animals was ensured before further processing and sampling. After the rams were slaughtered, fifteen pairs of testes and epididymis were recovered. The testes and epididymis were removed, cut into 1–2 cm cubes, placed in 10% neutral buffered formalin, and preserved for additional histopathological examination. The samples were cleaned, dried in ethyl alcohol in increasing concentrations, cleaned in methyl benzoate, and then embedded in paraffin wax. Hematoxylin and eosin were used to stain many paraffin slices, each 3–5 microns thick [27].

### Statistical analysis

JMP 13 software (SAS Institute Inc., Cary, NC, USA) was used for statistical analysis. JMP 13 was used to perform a one-way analysis of variance (ANOVA) and pooled t-tests on the data (SAS Institute Inc.). The P value was adjusted by comparing all pairs using the Tukey-Kramer HSD test. The MSW and HSW data at the end of the experiment (9 months) were compared to day 0 using Dunnett’s multiple comparisons procedure (pretreatment control). The correlations between the water salinity and sperm viability, morphology and sperm cell concentration were done using Spearman’s test. The values are shown as the mean standard error (SE). When the *p*-value was < 0.05, all differences were considered significant.

## Results

**Fig. 1:**
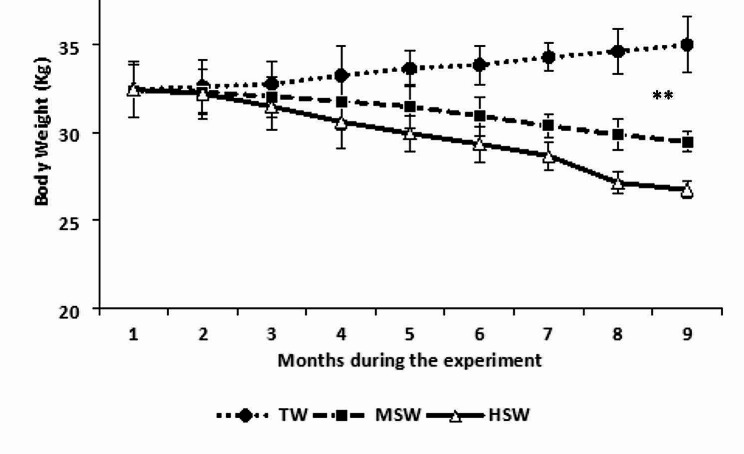
Rams live body weight during the 9 months of experiments in the HSW (high saline water; 8,934 ppm of total dissolved salts), MSW (moderate saline water; 4,557 ppm of total dissolved salts) and TW (control/tap water; 350 ppm of total dissolved salts) groups; ** significant at *P* < 0.05

At the start of the trial, the rams in the TW, MSW and HSW had body weights of 32.23 ± 0.01, 32.36 ± 0.02, and 32.27 ± 0.01, respectively (Fig. 1). When sheep were subjected to MSW and HSW, their body weight declined significantly (*p* < 0.05). However, TW grew slightly, which is not significant (*p* > 0.05; Fig. 1). Throughout the experiment, there was no mortality in all groups. Clinical data did not significantly differ between the groups during the experimental period.

**Fig. 2:**
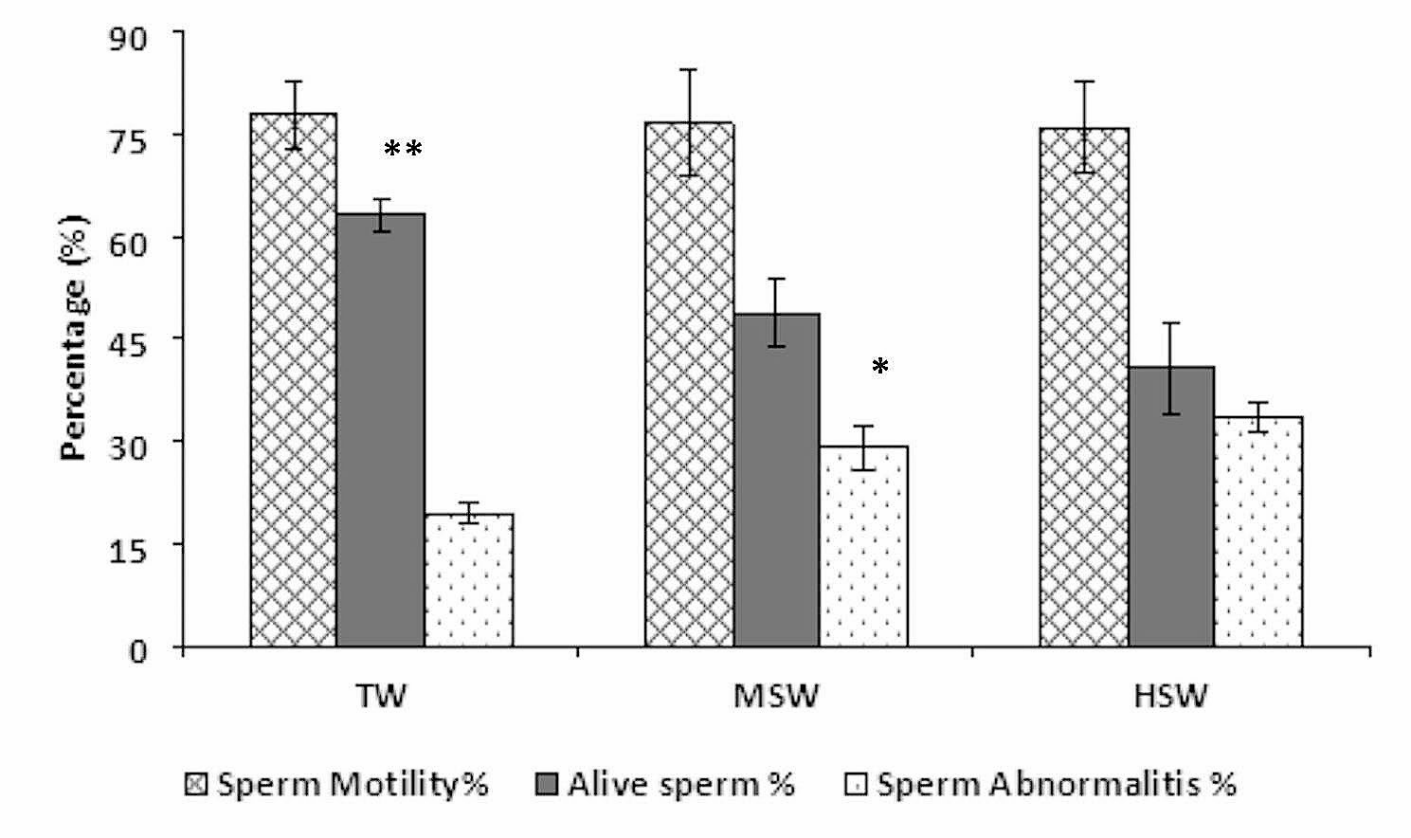
Percentages of sperm motility, viability (alive sperm), and sperm abnormalities in the HSW (high saline water; 8,934 ppm of total dissolved salts), MSW (moderate saline water; 4,557 ppm of total dissolved salts) and TW (control/tap water; 350 ppm of total dissolved salts) groups; ** significant at *P* < 0.05

**Fig. 3:**
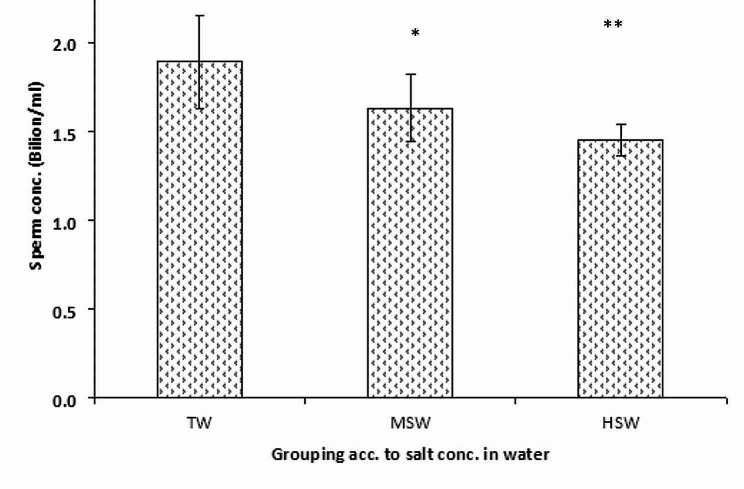
Sperm concentration × 10^9^ ; HSW (high saline water; 8,934 ppm of total dissolved salts); MSW (moderate saline water; 4,557 ppm of total dissolved salts); TW (control/tap water; 350 ppm of total dissolved salts) groups; ** significant at *P* < 0.05

The findings demonstrated no discernible difference in the mass sperm motility (∼ 3 scores) across the examined groups, MSW, HSW, TW, and the individuals (Fig. 2). In comparison to the TW group, the percentages of dead spermatozoa and aberrant sperms were considerably (*p* < 0.05) more significant in the HSW and MSD groups (Fig. 2). Sperm cell concentration (×109/ml) was negatively correlated (*p* < 0.05, *r* = -0.72) to the water salinity, where the number of spermatozoa decreased significantly (*p* < 0.05) in HSW than TW. The differences between MSW and TW were not significant (Fig. 3).

Sperm vitality and typical sperm morphology were negatively correlated with NO and MDA levels (*p* < 0.05, *r* = -0.85).

According to the results, the concentrations of nitric oxide (NO; Table 3) and malondialdehyde (MDA) were significantly higher in the MSW and HSW groups than they were in the TW (*p* < 0.05).

**Table 3:** Nitric oxide (NO), malondialdehyde (MDA); superoxide dismutase (SOD), glutathione (GSH), testosterone, LH and FSH concentrations in the HSW (high saline water; 8,934 ppm of total dissolved salts), MSW (moderate saline water; 4,557 ppm of total dissolved salts) and TW (control/tap water; 350 ppm of total dissolved salts) groups (Mean ± SEM, *n* = 6/each)

Parameter	HSW	MSW	Control	*p*-value
**NO (mmol/L)**	16.92 ± 0.51^a^	8.68 ± 0.49^b^	3.84 ± 0.50^c^	0.001
**MDA (nmol/L)**	591.25 ± 32.03^a^	275.20 ± 21.07^b^	120.62 ± 14.88^c^	0.001
**SOD (u/ml)**	155.22 ± 14.53^a^	258.67 ± 8.77^b^	418.63 ± 10.06^c^	0.01
**GSH (nmol/L)**	6.37 ± 0.23^a^	8.26 ± 0.24^b^	9.71 ± 0.36^c^	0.05
**cortisol (ug/dl)**	1.05 ± 0.06^a^	0.60.78 ± 0.04^b^	0.22 ± 0.03^c^	0.04
**testosterone (ng/ml)**	3.15 ± 0.51^a^	4.17 ± 0.26^b^	5.51 ± 0.29^c^	0.05
**LH (iu/ml)**	3.00 ± 0.25^a^	3.56 ± 0.17^ab^	4.97 ± 0.09^c^	0.05
**FSH (iu/ml)**	3.61 ± 0.27^a^	4.07 ± 0.12^ab^	5.76 ± 0.18^c^	0.05

Means with different superscripts (a, b,c) in the same rows are sig. differ (*P* < 0.05)

Control group offered tap water with 350 ppm TDS, Moderate saline water group (MSW offered water with 4,557 ppm TDS, High saline water group (HSW) offered water with 8,934 ppm TDS.

While there was a significant (*p* < 0.05) decrease in glutathione (GSH) concentration as well as superoxide dismutase activity (SOD) in both the moderate and high groups compared to the control group. Compared to the control group, the HSW group had the greatest cortisol hormone levels, followed by the MSW group (Table 3). The testosterone, LH, and FSH concentrations in the HSW and MSW groups were noticeably lower than those in the control group, and the differences were statistically significant (*p* < 0.05).

**Fig. 4:**
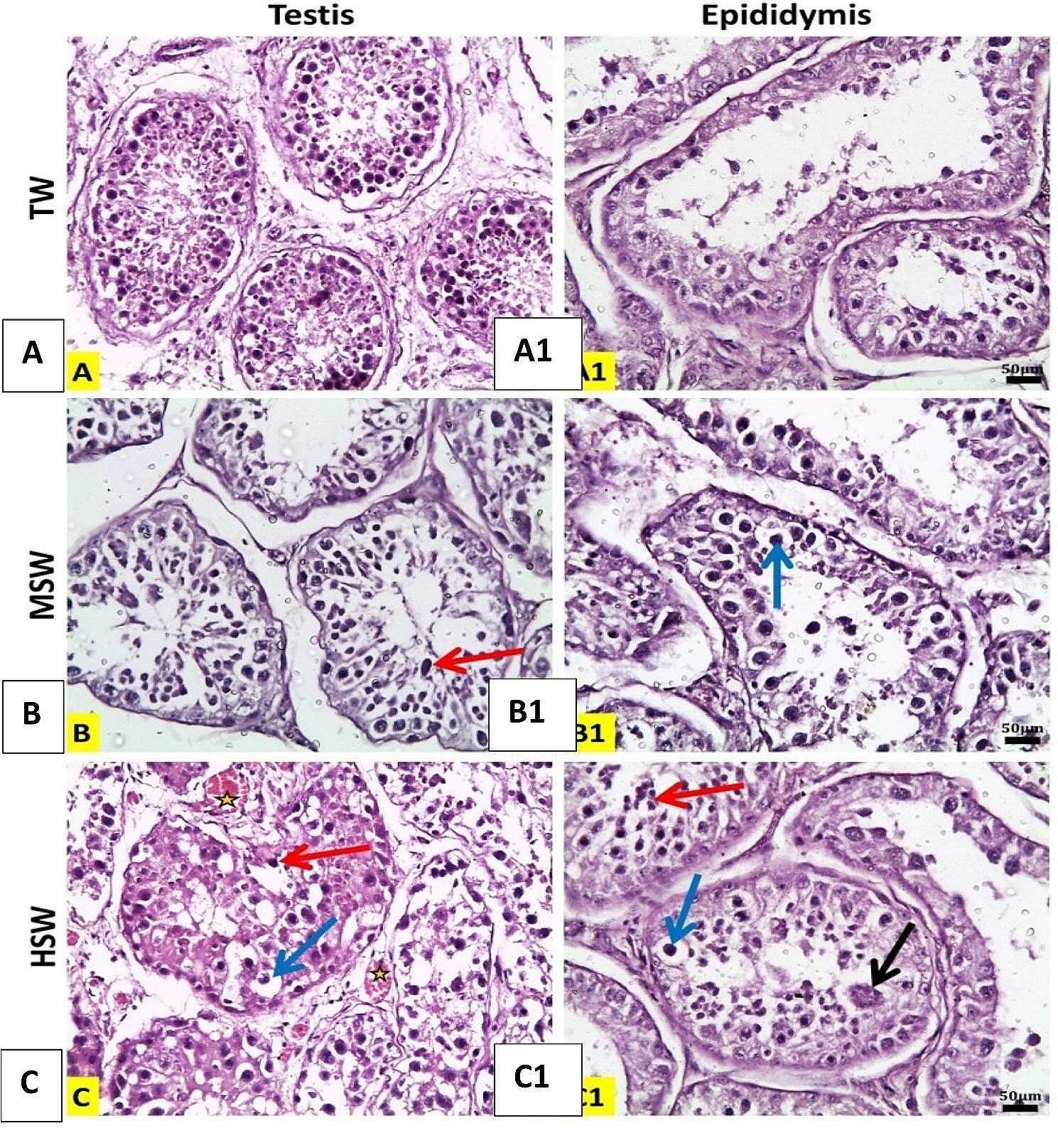
Histopathological photomicrographs of ram testes (A, B, C) & tail of the epididymis (A1, B1, C1) sections of different groups (Haematoxylin and Eosin stain); X20, Scale bar 50 μm. Star: congestion, black arrows: giant cell, blue arrows: vacuolated lining epithelial cells with pyknotic nucleus, red arrows: desquamated necrotic cells. (A, A1)

Barki Rams in the TW group display normal testis and epididymis histological appearances. (Fig. 4): The MSW group demonstrating vacuolation of lining epithelial cells with pyknotic nuclei in the epididymis and necrosis and desquamation of spermatogenic cells in seminiferous tubules (Fig. 4): The HSW group displaying desquamated necrotic cells and giant cell formation in the epididymis, as well as damage to some of the seminiferous tubules and showed congestion, vacuolation of spermatogenic epithelium of seminiferous tubules, and desquamated necrotic spermatogenic epithelium (Fig. 4).

## Discussion

There is insufficient information on how high salt levels affect male fertility. This study set out to investigate the effects of excessive salt content in rams’ drinking water on body weight, oxidative stress, the concentration of the hormones FSH, LH, and cortisol, and testicular histopathology in the local Egyptian desert environment. In general, the results were remarkably different in the high-water salinity group and were consistent with the earlier results [28].

Our findings showed substantial differences between the control group and all evaluated parameters (drinking tap water). Nitric oxide (NO) concentration was highest in the HSW group, followed by the MSW group, and lowest in the TW group, which may have impaired genital tract blood supply and adversely affected the process of spermatogenesis. In experimental studies, it was discovered that, in the unilateral testicular torsion group, NO content, NOS activity, and pathological scores of the contralateral testis all increased to varying degrees and that NO content was positively correlated with pathological score, indicating that the more NO concentration, the more tissue injury. By combining NO with oxygen-free radicals on the torsional side, the injury may result in additional harmful ONOO-, which could harm the testicular tissue on the opposite side. Alternatively, it might accumulate pro-inflammatory NO transmitters in the contralateral testicular tissue, enlarge blood vessels excessively, and trigger apoptosis [29]. The fact that the injury above can simultaneously lead to further NOS activation, which can produce more NO and result in more damage, shows that the effect of NO on reproductive tissues is dose-dependent. Some disorders are also linked to NO. For instance, the iNOS subtype stimulates excessive NO production in varicocele in patients with varicocele, which can result in impaired sperm motility and even sterility [30]. The control of sperm motility, hyperactivation during capacitation, and fertilization appears to be significantly influenced by NO [31].

NO’s effects on sperm viability and morphology show conflicting contributions. Males with average sperm viability rates of under 14% have a positive association between NO and faults in sperm morphology. Still, males with average sperm morphology rates of under 14% have a negative correlation [32]. However, the following research failed to discover any meaningful connection between NO generation and sperm morphology [33]. The present investigation showed no discernible differences in sperm motility across the examined groups, MSW, HSW, and TW. The aberrant and dead spermatozoa percentages were significantly more significant in the HSW and MSD groups than in the TW group, and these results were consistent with earlier data [28]. It was noticed that high salt concentration (HSW) adversely affected the sperm cell concentration compared to that in the MSW and TW groups. Similar results were observed in rats, with a negative association between NO and MDA levels and sperm vitality and normal sperm morphology (P 0.05, *r* = -0.85) [32].

Malondialdehyde (MDA) concentrations were significantly higher in the MSD and HSW groups than in the control group in the current study. Malondialdehyde (MDA) is an essential indicator of lipid peroxidation in cells. When compared to controls, mice fed high salt diets (8%) had significantly higher MDA levels and superoxide dismutase (SOD) activities in the testis [34]. It was found that rats given 8% NaCl had higher MDA levels in the epididymis than controls, consistent with previous research. The elevated levels of MDA and SOD seen in the testis of rats given a high-sodium diet can be attributable to free radical damage, supporting the harmful effects of a high-sodium diet on reproductive functions [35]. Antioxidant enzymes are typically created in response to elevated levels of free radicals; however, if high levels of oxidative stress disrupt antioxidant enzymes, they can be detrimental to reproduction. However, it was discovered in the current study that both superoxide dismutase activity (SOD) and glutathione (GSH) concentrations were significantly lower in the moderate and high salt groups than in the control group. Similarly, Iranloye et al. [35] discovered that increased dietary salt in rats dramatically decreased the activity of SOD, GSH, and CAT enzymes in testicular and epididymal tissues. In contrast to the present and the earlier findings [34], It was found that in mice, the levels of GSH and catalase were not substantially different between the high salt and control groups; this discrepancy could be attributed to the salt concentrations and species variations.

According to studies, some forages, including *Suaeda glauca*, have a salt level of up to 317–331 g/kg dry matter (DM), while many other forages have a salt content of 15–41 g/kg DM, which is higher than the salt content of 1.2–1.8 g/kg DM in most forages [28]. Additionally, up to 0.9–1.7% of salt is present in surface water in saline places, which exacerbates the increased salt consumption [36]. Numerous animal problems, including lipid buildup, kidney injury, hypertension, and failure of renal function because of excessive salt intake, have been documented [37]. Low fertility and sperm dysfunction are both symptoms of oxidative stress in animals [38, 39].

In our study, the HSW group had the greatest cortisol hormone concentration, followed by the MSW group, while the control group had the lowest cortisol hormone concentration. Compared to the control group, there were significant drops in the levels of LH, FSH, and testosterone (T) in the HSW and MSW groups. Numerous studies have shown the effects of increased salt intake on hormone balance. The feeding of rams with high salt diets resulted in significant reductions of leptin, testosterone (T), insulin, luteinizing hormone (LH), and follicle-stimulating hormone (FSH) levels (12%) in comparison to controls [28]. Leptin is well known to play an essential role in signalling nutritional status in the mammalian centrally reproductive axis, and it is also thought to be a vital factor for puberty initiation, as it induces GnRH and LH release [40]. The decrease in leptin and insulin could be attributed to a reduction in body mass and a decline in fat reserves. These hormones also regulate metabolism, homeostasis, and body mass [41].

Additionally, a 20% salt diet of lambs decreased their energy metabolism and peripheral insulin levels [42]. According to a study, excessive salt intake lowers leptin and insulin levels without affecting feed intake (FI) or body weight gain (BWG) [43]. Several earlier investigations have described endocrine abnormalities in animals fed a high-sodium diet, where it was found that FSH, LH, and T plasma concentrations had significantly decreased [28, 44]. As a result of the influence of a high salt diet, corticosterone levels were seen to increase significantly, and T levels noticeably decreased in rats [35]. On the other hand, it was found that increased salt in the diet (8%) compared to control rats did not affect LH and FSH levels [35]. A high-salt diet may decrease T production, impacting spermatogenesis and changing sperm quality. It was also found that, despite the low T level, the gonadotropin level in response to LH stimulation was not significantly impacted. High salt inhibited the hypothalamic-pituitary axis by interacting with the Leydig cells’ LH receptors [35]. The enzyme 11betahydroxysteroid dehydrogenase type 1 (11-HSD1) has also shown that high sodium consumption increases the secretions of glucocorticoid hormones [45, 46]. It is generally known that dietary stress causes a rise in corticosterone.

Furthermore, high salt intake impacts reproductive function because it can lower fertility due to elevated cortisol levels [47]. One study reported that when rats were fed a high-salt diet, FSH and testosterone levels significantly increased, whereas LH levels decreased compared to controls [36]. There is still work to be done on this problem. Generally, a diet heavy in salt reduces fat storage in the body, lowering the quantity of cholesterol required to produce sexual hormones. As a result, decreased spermatogenesis, a factor in lower reproductive capability, was caused by a decrease in the synthesis of sex hormones like testosterone.

It was found that Leydig cell loss or damage can result in the critical spermatogenesis genes being downregulated [48]. Examining the reproductive health of rats on a high-salt diet found altered testicular morphology, altered gene expression related to sperm quality in males, lower testicular weight, and impaired sperm function [28].

In the MSW group, histopathological findings included necrosis and desquamation of spermatogenic cells in seminiferous tubules and vacuolation of lining epithelial cells with pyknotic nuclei in the epididymis. The HSW group’s findings revealed damage to nearly all seminiferous tubules, including congestion and vacuolation, as well as desquamation of the necrotic spermatogenic epithelium and giant cell formation in the epididymis. It was reported before that the high salt group of rams had a higher percentage of apoptotic cells [49]. Apoptosis is unquestionably essential, and an increase or decrease in apoptosis rate during the spermatogenesis process can result in defects in spermatogenesis [50, 51]. In a previous study, the higher number of TUNEL-positive cells found in the treatment with the most elevated salinity (8326 mg/mL TDS) may also have contributed to the abnormalities in sperm, which would have affected the development of spermatozoa. The cells may be impacted by a shift in the extracellular ion concentration, which could lead to Ca2 + channel changes, oxidative stress, and increased apoptosis [52].

## Conclusion

High salt concentration in drinking water adversely affected sperm viability, morphology and sperm cell concertation. The antioxidants biomarkers were significantly decreased after consumption of water high in salt content, on the other hand the concentration of nitric oxide and malondialdehyde increased by consumption saline water. The concentration of stress hormone (cortisol) showed marked increase, while FSH, LH as well as Testosterone concentrations were remarkably low in groups consumed saline water. High salt concentration in drinking water could increase damage to testicular tissue in Barki rams, decrease hormonal concentrations which primarily included in reproduction control as FSH, LH and T. Further studies are required to confirm these findings.

## Data Availability

No datasets were generated or analysed during the current study.
